# Navigating single-cell RNA-sequencing: protocols, tools, databases, and applications

**DOI:** 10.1186/s44342-025-00044-5

**Published:** 2025-05-17

**Authors:** Ankish Arya, Prabhat Tripathi, Nidhi Dubey, Imlimaong Aier, Pritish Kumar Varadwaj

**Affiliations:** Department of Applied Sciences, Indian Institute of Information Technology Allahabad, Jhalwa, Prayagraj, 211015 Uttar Pradesh India

**Keywords:** Single-cell RNA sequencing, Databases, Protocols and tools, Cellular heterogeneity, Drug discovery

## Abstract

Single-cell RNA-sequencing (scRNA-seq) technology brought about a revolutionary change in the transcriptomic world, paving the way for comprehensive analysis of cellular heterogeneity in complex biological systems. It enabled researchers to see how different cells behaved at single-cell levels, providing new insights into the process. However, despite all these advancements, scRNA-seq also experiences challenges related to the complexity of data analysis, interpretation, and multi-omics data integration. In this review, these complications were discussed in detail, directly pointing at the optimization of scRNA-seq approaches and understanding the world of single-cell and its dynamics. Different protocols and currently functional single-cell databases were also covered. This review highlights different tools for the analysis of scRNA-seq and their methodologies, emphasizing innovative techniques that enhance resolution and accuracy at a single-cell level. Various applications were explored across domains including drug discovery, tumor microenvironment (TME), biomarker discovery, and microbial profiling, and case studies were discussed to explain the importance of scRNA-seq by uncovering novel and rare cell types and their identification. This review underlines a crucial aspect of scRNA-seq in the advancement of personalized medicine and highlights its potential to understand the complexity of biological systems.

## Introduction

Two centuries after Robert Hooke and Antonie van Leeuwenhoek, cells were redefined as the fundamental functional unit of life [1]. Since then, researchers have conducted numerous experiments and developed various techniques to study cells within complex multicellular systems for a more comprehensive understanding [2, 3]. Over the past decade, bulk RNA-sequencing technologies have been widely employed to investigate gene expression patterns on a population scale, which allowed researchers to analyze the transcriptome of a group of cells or tissues, providing insights into gene activity levels within the sample. The emergence of single-cell RNA-sequencing opened up remarkable prospects for investigating gene expression profiles at the individual cell level, when scRNA-seq was first reported in the 4-cell blastomere stage in the year of 2009. Consequently, in 2014, the first multiplexed scRNA-seq method was developed [4, 5]. In 2017, scRNASeqDB, a database dedicated to gene expression profiles for human single cells, was created [6]. In 2021, Asc-Seurat, a user-friendly web application for comprehensive scRNA-seq data analysis, was developed, which can perform complete analysis [7].

At present, scRNA-seq is increasingly being preferred when addressing crucial biological inquiries related to cell heterogeneity and early embryo development, particularly in cases involving a limited number of cells. In recent times, scientists have used scRNA-seq on a wide range of species, particularly in various human tissues, both healthy and cancerous. These studies have revealed that gene expression can differ significantly from one cell to another. This insight helps understand how genes are active or inactive in individual cells, shedding light on the complexity of our biology [8, 9]. ScRNA-seq is a powerful technique for tackling issues related to the unpredictable behavior of genes. Currently, studies using scRNA-seq hold significant potential for uncovering previously unknown cell types, mapping out developmental pathways, and investigating the complexity of tumor diversity [10]. The key contrast between bulk RNA-seq and scRNA-seq is whether each library reflects an individual cell or a cell group, driven by challenges like scarce transcripts in single cells, inefficient mRNA capture, losses in reverse transcription, and bias in cDNA amplification due to the minute amounts involved [11, 12]. During quality control, when using scRNA-seq, it is important to identify and remove low-quality individual cells and any data that might represent multiple cells. In some methods like drop-based sequencing, background noise can also be removed. However, one should be careful when applying data normalization techniques designed for bulk RNA-sequencing because they can introduce errors into scRNA-seq data [13]. When it comes to aligning sequencing data, the tools commonly used for bulk RNA-sequencing can also be used for scRNA-seq data. However, dedicated alignment methods designed for scRNA-seq often offer advantages in terms of efficient use of computing resources and faster processing speed [11, 14]. There are often missing values in scRNA-seq data. To combat this issue, multiple imputation algorithms which rely on various models can be employed. When dealing with batch effects in scRNA-seq, it is crucial to account for both technical deviations and biological differences. The goal is to preserve the biological variation of interest by reducing unwanted variation. While scRNA-seq provides valuable advantages for biological research, it has notable limitations. Gene expression data obtained through this process is often noisy, high-dimensional, and sparsely populated. Consequently, to fully harness the potential of scRNA-seq technology, specialized computational tools tailored to scRNA-seq data are essential [11]. In recent years, the explosion of single-cell analysis tools has increased the difficulty of selecting the right tool for a given dataset [15, 16]. Many tools are designed to simplify the processing and comprehension of scRNA-sequencing data through user-friendly interfaces [17, 18]. Nevertheless, to the uninitiated, these tools and algorithms can resemble elusive “dark elixirs.” Empowering researchers in their quest for the most fitting methods, algorithms, and tools demands a comprehensive review that unveils the inner workings of these computational marvels.

## Different protocols for scRNA-seq: an overview

Many scRNA-seq approaches have been suggested for single-cell transcriptomic research (Table 1). After the initial scRNA-seq technique was published, several alternative scRNA-seq strategies emerged. These scRNA-seq technologies differ in at least one of the following areas: availability of Unique Molecular Identifiers (UMIs), cell isolation, cell lysis, reverse transcription, amplification, transcript coverage, and transcription. One obvious distinction between these scRNA-seq approaches is that some of these techniques can generate full-length (or nearly full-length) transcript sequencing data (e.g., Sn-drop, Smart-Seq2, Quartz-Seq2, MATQ-Seq, and Fluidigm C1), whereas others can only capture and sequence the transcripts 3′ or 5′ ends (e.g., REAP-Seq, Drop-Seq, inDrop, Seq-Well, DroNC-Seq, and SPLiT-Seq). Different scRNA-seq techniques each have unique benefits and restrictions. Numerous evaluations that have been published analyze thorough comparisons. According to one research, Smart-Seq2 performs better than other scRNA-seq technologies, including CEL-Seq2, MARS-Seq, Smart-Seq, and Drop-Seq procedures, in identifying more expressed genes. Furthermore, MATQ-Seq is superior to Smart-Seq2 in detecting low-abundance genes. Full- length scRNA-Seq methods offer unique advantages over 3′ end or 5′ end counting protocols. They excel in tasks like isoform usage analysis, allelic expression detection, and identifying RNA editing due to their comprehensive coverage of transcripts. Furthermore, in the detection of specific lowly expressed genes or transcripts, full-length scRNA-seq approaches may outperform 3′ end sequencing methods [19]. Droplet-based techniques like Drop-Seq, InDrop, and Chromium often enable a higher throughput of cells and a lower sequencing cost per cell as compared to whole-transcript scRNA-seq [20–22]. The ability to handle large numbers of cells makes droplet-based techniques particularly helpful for detecting various cell subpopulations inside complex tissues or tumor samples. While inDrop and CEL-Seq2 rely on in vitro transcription (IVT) for amplification, the remaining protocols utilize polymerase chain reaction (PCR) as their amplification method as described in Table 1.

**Table 1:** Comparison of protocols based on isolation strategy, transcript coverage, UMI usage, and amplification method

Protocols	Isolation strategy	Transcript coverage	UMI	Amplification method	Unique features
SnDrop [23]	Droplet-based	Full-length	Yes	PCR	Combines nuclei isolation with droplet microfluidics; reduces dissociation artifacts
REAP-Seq [24]	Droplet-based	3′-only	Yes	PCR	Allows simultaneous protein and RNA detection
Smart-Seq2 [25]	FACS	Full-length	No	PCR	Enhanced sensitivity for detecting low-abundance transcripts; generates full-length cDNA
Drop-Seq [26]	Droplet-based	3′-end	Yes	PCR	High-throughput and low cost per cell; scalable to thousands of cells simultaneously
inDrop [27]	Droplet-based	3′-end	Yes	IVT	Uses hydrogel beads; low cost per cell; efficient barcode capture
STRT-Seq [28]	FACS	5′-only	Yes	PCR	High-resolution mapping of transcription start sites
CEL-Seq2 [29]	FACS	3′-only	Yes	IVT	Linear amplification reduces bias compared to PCR
Seq-well [30]	Droplet-based	3′-only	Yes	PCR	Portable, low-cost, easily implemented without complex equipment
Quartz-Seq2 [31]	FACS	Full-length	No	PCR	Optimized reaction conditions for improved sensitivity
DroNC-Seq [32]	Droplet-based	3′-only	Yes	PCR	Specialized for single-nucleus sequencing, minimal dissociation bias
sci-RNA-Seq [33]	FACS	3′-only	Yes	PCR	Combinatorial indexing for ultra-high throughput without single-cell isolation equipment
SPLiT-Seq [9, 34]	Not required	3′-only	Yes	PCR	Combinatorial indexing without physical separation; highly scalable and low cost
MATQ-Seq [35]	Droplet-based	Full-length	Yes	PCR	Increased accuracy in quantifying transcripts; efficient detection of transcript variants
Fluidigm-C1 [36]	Droplet-based	Full-length	No	PCR	Microfluidics-based single-cell capture; precise cell handling

## Currently employed methodologies

**Fig. 1:**
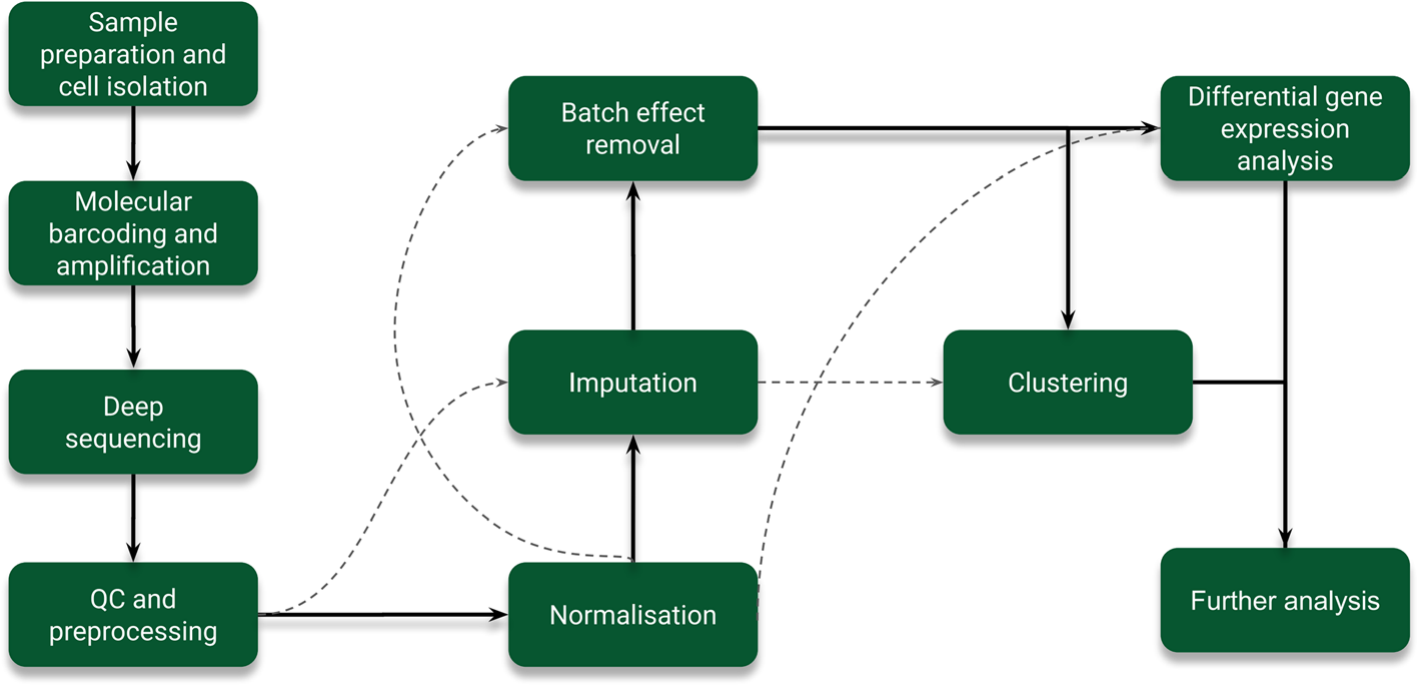
A graphical overview of the steps involved in scRNA sequencing. There are certain tools that skip certain steps: DeepImpute and MAGIC skip normalization to perform batch effect correction, while EdgeR, MAST, and Monocle2 skip imputation in order to perform differential gene expression analysis. DESeq2 directly bypasses normalization and imputation to perform differential gene expression. Furthermore, clustering is done immediately by RaceID, SC3, and Monocle2 without the need for batch effect correction

The primary steps involved in scRNA-seq encompass single-cell isolation and capture, cell lysis, reverse transcription, cDNA amplification, and library preparation (Fig. 1).

### Sample preparation and cell isolation

The initial stage of performing scRNA-seq involves the extraction of viable and individual cells from the specific tissue under investigation. Novel methodologies, such as the isolation of individual nuclei for RNA-seq (snRNA-seq), are used in conditions where tissue dissociation is challenging, or when samples are frozen or cells are fragile. Other methodologies include the use of “split-pooling” scRNA-seq techniques, which apply combinatorial indexing (cell barcodes) to single cells, offering distinct advantages over the isolation of intact single cells. These advantages include the ability to handle large sample sizes (up to millions of cells) and greater efficiency in parallel processing of multiple samples while eliminating the need for expensive microfluidic devices. Subsequently, the individual cells are subjected to lysis in order to facilitate the capture of RNA molecules. Poly[T]-primers are frequently employed to selectively analyze polyadenylated mRNA molecules while minimizing the capture of ribosomal RNAs [37]. Table 2 gives an overview of different methods for cell-cell preparation and isolation strategy.

**Table 2:** Summary of different methods of cell preparation and isolation strategy

Method	Principle	Advantages	Limitations	Applications
FACS [38]	Fluorescence-based sorting using specific cell markers	Highly selective, precise isolation	Expensive, cellular stress	Targeted cell populations
Microfluidics (droplet-based) [39]	Encapsulation of cells in droplets with barcoded beads	High-throughput, efficient, automated	High cost, transcript loss	Large-scale profiling, general use
Split-pooling [40]	Combinatorial barcoding without physical isolation	Cost-effective, highly scalable	Complex data handling, barcode collisions	Large-scale studies, multiplexed samples
snRNA-seq [41]	Isolation of nuclei instead of intact cells	Minimal dissociation stress, suitable for frozen tissues	Lower RNA yield, excludes cytoplasmic transcripts	Difficult to dissociate tissues, archival samples

### Molecular barcoding and amplification

Following the conversion of RNA into complementary DNA (cDNA), the resulting cDNA molecules undergo amplification by either the polymerase chain reaction (PCR) or in vitro transcription (IVT) methods. PCR, a non-linear amplification process, is utilized in several methodologies such as Smart-Seq, Smart-Seq2, Fluidigm C1, Drop-Seq, 10x Genomics, MATQ-Seq, Seq-Well, and DNBelab C4. At present, there are two distinct techniques for PCR amplification.

The utilization of SMART technology involves the exploitation of the transferase and strand-switch activity of Moloney murine leukemia virus reverse transcriptase. This enzyme is employed to integrate template-switching oligos as adaptors for subsequent PCR amplification. The aforementioned approach was widely employed for the amplification of cDNA. The alternative approach involves the ligation of common adaptors to the 5′ end of cDNA, using either poly(A) or poly(C), in order to facilitate the subsequent PCR reaction. The IVT method is a technique employed in many sequencing procedures like as CEL-Seq, MARS-Seq, and inDrop-Seq. It serves as an amplification strategy and facilitates linear amplification of genetic material. A second iteration of reverse transcription of the amplified RNA is necessary, leading to the emergence of further 3′ coverage biases. Both methodologies have the potential to result in amplification biases. In order to mitigate biases associated with amplification, a technique called UMIs was implemented. UMIs are used to label each individual mRNA molecule within a cell during the reverse transcription process. This approach enhances the quantitative aspect of scRNA-seq and improves the accuracy of data interpretation by effectively eliminating biases introduced by PCR amplification. The CEL-Seq, MARS-Seq, Drop-Seq, inDrop-Seq, 10x Genomics, MATQ-Seq, Seq-Well, and DNBelab C4 methods have incorporated UMIs [3].

### Deep sequencing

After the generation of single cell-barcoded cDNAs from individual cells or nuclei, the subsequent sequencing of the cDNA can be performed using several advanced sequencing technologies. Regarding high-throughput sequencing using DNA nanoballs (DNBseq), the DNA fragments chosen were subjected to repair processes to achieve a blunt end and were subsequently changed at the three ends to generate a dATP overhang. Following this, the dTTP-tailed adapter sequence was utilized to ligate each end of the DNA fragment. The ligation result was subsequently subjected to a few cycles of amplification, followed by a single-strand cycle. A specific segment of the PCR product was subjected to reverse complementation using a specialized molecule, followed by ligation with a single-stranded molecule using DNA ligase. This process ultimately resulted in the generation of a circular DNA library consisting of single-stranded molecules [3]. Some other sequencing platforms which are relevant to scRNA-seq are Illumina Sequencing (short-read sequencing), DNBseq (DNA Nanoball sequencing, short-read sequencing), Oxford Nanopore Technologies (long-read sequencing), and Pacific Biosciences (PacBio, single molecule real-time sequencing) [42]. Table 3 gives a comparative overview of sequencing platforms.

**Table 3:** Comparison of sequencing platforms based on key parameters

Sequencing platform	Principle	Throughput	Read length	Accuracy	Cost	Error type	Application
Illumina [43]	SBS	Very high	Short (50–300 bp)	Very high ($\sim$99.9%)	Cost-effective	Substitutions	High-throughput gene quantification
DNBSeq [3]	DNA Nanoball sequencing	Very high	Short (50–200 bp)	Very high ($\sim$99.9%)	Cost-effective	Substitutions	High-throughput gene quantification
Oxford Nanopore [44]	Nanopore sequencing	Moderate–high	Long (up to > 100 kb)	Moderate (90–98%)	Moderate, declining	Indels/substitutions	Full-length isoform detection
Pacific Biosciences [44]	Single molecule real-time (SMRT)	Moderate–low	Long (5–30 kb or more)	Moderate to very high (90–99.9%)	Higher, declining	Indels/substitutions	Isoform characterization and full-length transcript profiling

### Quality check and pre-processing

Performing quality control is important to remove consistent technical variations that might have been introduced in generating the data, to focus on the biological variations of cells. The random sampling procedure and the limited RNA content in data increase noise, compared to bulk RNA-sequencing [45–47]. Dropout events (genes not detected due to low RNA content) create excessive zero counts [48] and can lead to highly sparse datasets, making it difficult to detect genuine biological variations within individual cells. Filtering out lower quality cells is crucial for expression analysis; criteria vary based on cell and tissue type [3, 20, 49].

Two primary quality metrics in scRNA-seq are expressed counts and total library size. Low counts suggest poor RNA capture, while high counts may indicate multiple cells captured erroneously, depicted visually via violin plots [17, 19, 50]. Filtering out potential doublets or multiplets in scRNA-seq involves setting thresholds based on expressed feature counts, affected by both biological and technical factors. Sequencing depth influences read and feature counts, and using robust statistics like median absolute deviations aids in identifying outlier cells [51–53]. There are certain established methods for detecting doublets [54], which are given in Table 4. Mitochondrial gene presence in the data can also affect the quality of data; its proportion can vary from tissue to tissue like heart cells typically exhibit around 30%, contrasting with lymphocytes [55, 56].

**Table 4:** List of tools used in scRNA-seq analysis

Tool name	Input format	Algorithm framework	Output format	Specific use case	Language	Availability
**Pre-processing and quality control**						
FastQC [57]	FASTQ	Identifies overrepresented short sequences (k-mers)	HTML, ZIP	NA	JAVA	https://www.bioinformatics.babraham.ac.uk/projects/fastqc/
PopsicleR [58]	Expression matrix (e.g., CSV, TSV)	Uses a mixture model of mitochondrial content and gene expression levels to classify cells as low or high-quality	Filtered expression matrix, plots (e.g., PDF, PNG)	Suitable for detecting rare cell populations	R	https://github.com/bicciatolab/popsicleR
miQC [59]	Expression matrix, metadata (e.g., CSV)	Uses probabilistic approach to identify empty droplets or droplets with low RNA content by modeling the distribution of RNA molecules per droplet	Filtered expression matrix, plots (e.g., PNG)	Mitochondrial contamination assessment	R	https://www.bioconductor.org/packages/release/bioc/html/miQC.html
DropletQC [60]	Expression matrix (e.g., CSV, TSV)	Uses supervised learning algorithms to detect and classify doublets by comparing observed gene expression with simulated doublets	Quality-controlled data (e.g., CSV, HDF5)	Identifies empty droplets and low-quality cells in droplet-based scRNA-seq data	R	https://github.com/powellgenomicslab/DropletQC
Solo [61]	Expression matrix, BAM	Uses supervised learning algorithms to detect and classify doublets by comparing observed gene expression with simulated doublets	Filtered expression matrix, summary stats	Efficiently remove doublets	Python	https://docs.scvi-tools.org/en/stable/user_guide/models/solo.html
Trim Galore [62]	FASTQ	Uses Cutadapt for adapter trimming and FastQC for quality control	Trimmed FASTQ	NA	Python	https://github.com/FelixKrueger/TrimGalore
DecontX [63]	Expression matrix	Bayesian hierarchical model	Decontaminated count matrix	Removes ambient RNA contamination	R	https://bioconductor.org/packages/release/bioc/html/decontX.html
CellBender [64]	Count matrices (HDF5)	Variational inference, deep learning	Cleaned count matrix	Droplet-based scRNA-seq ambient RNA removal	Python	https://github.com/broadinstitute/CellBender
Scater [65]	Single-cell expression matrix (e.g., CSV, TSV)	Uses threshold-based filtering and visualization to assess quality metrics	Quality-controlled expression matrix, plots (e.g., PNG, PDF)	NA	R	https://bioconductor.org/packages/release/bioc/html/scater.html
Seurat [49]	Expression matrix (e.g., CSV, TSV)	Uses heuristic filtering based on gene count, mitochondrial content, and feature expression to remove low-quality cells	Filtered and normalized data, plots, R objects	NA	R	https://satijalab.org/seurat/
Scanpy [50]	Expression matrix (e.g., CSV, TSV, H5 AD)	Uses various metrics like gene count, mitochondrial gene percentage, and UMI count for filtering	Processed AnnData (H5 AD), plots	NA	Python	https://scanpy.readthedocs.io/en/stable/
Scrublet [52]	Expression matrix (e.g., CSV, TSV)	Uses a k-NN classifier to detect doublets by comparing observed gene expression profiles with simulated doublets	Doublet scores, data Filtered	Detects doublets	Python	https://github.com/swolock/scrublet
ScPipe [66]	FASTQ, BAM, GTF	Uses a series of filtering steps based on read alignment quality, gene count, and expression levels to remove low-quality cells	Processed expression matrix (e.g., CSV, HDF5)	NA	R	https://github.com/LuyiTian/scPipe
Doublet Finder [67]	Expression matrix (e.g., CSV, TSV)	Machine learning approach to identify doublets by creating artificial doublets and comparing them with observed data	Doublet scores, data filtered	Doublet detection	R	https://github.com/chris-mcginnis-ucsf/DoubletFinder
SoupX [68]	Expression matrix (e.g., CSV, TSV)	Bayesian model to estimate and subtract ambient RNA contamination	Corrected expression matrix (e.g., CSV, HDF5)	Corrects ambient RNA contamination in droplet-based dataset	R	https://github.com/constantAmateur/SoupX
**Normalization tools**						
Dino [69]	Expression matrix (e.g., CSV, TSV)	Uses a Bayesian latent variable model to estimate and remove technical noise from gene expression data	Normalized expression matrix	Designed to improve signal recovery and account for technical variations	Python	https://www.bioconductor.org/packages/release/bioc/html/Dino.html
Scran [70]	Expression matrix (e.g., CSV, TSV)	Uses a deconvolution approach to compute size factors for normalization, adjusting for cell-specific biases	Normalized expression matrix, size factors	Effective for addressing cell-specific biases	R	https://www.bioconductor.org/packages/release/bioc/html/scran.html
Seurat [49]	Expression matrix (e.g., CSV, TSV)	Uses log-normalization, where counts are divided by total counts per cell and multiplied by a scaling factor, followed by log transformation	Normalized expression matrix, R objects	Provides multiple normalization methods	R	https://satijalab.org/seurat/
Scanpy [50]	Expression matrix (e.g., CSV, TSV, H5 AD)	Supports multiple methods including log-normalization, total count scaling, and variance-stabilizing transformations	Normalized AnnData (H5 AD)	Provides multiple normalization methods	Python	https://scanpy.readthedocs.io/en/stable/
DESeq2 [71]	Count matrix (e.g., CSV, TSV)	Uses a median of ratios method to normalize counts, adjusting for differences in sequencing depth and RNA composition between cells	Normalized count matrix, DE results (CSV, TSV)	NA	R	https://bioconductor.org/packages/release/bioc/html/DESeq2.html
SCnorm [72]	Expression matrix (e.g., CSV, TSV)	Uses a quantile regression approach to normalize gene expression, correcting for gene-specific biases across cells	Normalized Expression Matrix	Address gene-specific biases in expression levels	R	https://bioconductor.org/packages/release/bioc/html/SCnorm.html
LIGER [73]	Expression matrix (e.g., CSV, TSV, HDF5)	Uses iNMF to normalize and integrate data across different datasets, enabling joint analysis of multiple datasets	Integrated and normalized matrix	Harmonize multiple dataset	R	https://github.com/welch-lab/liger
**Imputation tools**						
IGSimpute [74]	Expression matrix (e.g., CSV, TSV)	Uses a graph-based method, where gene similarities are calculated, and missing values are imputed based on similar genes’ expression patterns	Imputed expression matrix	Preserving gene-gene relationships	R	https://github.com/ericcombiolab/IGSimpute
SciGAN [75]	Expression matrix (e.g., CSV, TSV)	Uses GANs to model the distribution of observed data and impute missing values by generating realistic data points	Imputed expression matrix	Maintains biological variability	Python	https://github.com/xuyungang/scIGANs
CMF-Impute [76]	Expression matrix (e.g., CSV, TSV)	Uses coupled matrix factorization, where data from multiple related matrices is factorized simultaneously to estimate missing values	Imputed expression matrix	NA	R	https://github.com/xujunlin123/CMFImpute
scIMC [77]	Expression matrix (e.g., CSV, TSV)	Uses IMC learning to impute missing values by leveraging shared information across multiple cell clusters	Imputed expression matrix	NA	Python	https://server.wei-group.net/scIMC/
DrImpute [78]	Expression matrix (e.g., CSV, TSV)	Uses clustering-based imputation where similar cells are grouped, and missing values are estimated based on the expression of similar cells	Imputed expression matrix	NA	R	https://github.com/gongx030/DrImpute
Deep Impute [76]	Expression matrix (e.g., CSV, TSV)	Uses deep neural networks to learn the underlying data structure and impute missing values	Imputed expression matrix	NA	Python	https://github.com/lanagarmire/deepimpute
ScLRTC [79]	Expression matrix (e.g., CSV, TSV)	Uses LRTC, where the data is modeled as a tensor, and missing values are imputed by completing the low-rank tensor	Imputed expression matrix	NA	R	https://github.com/jianghuaijie/scLRTC
ScHinter [80]	Expression matrix (e.g., CSV, TSV)	Uses hierarchical Bayesian models to impute missing data, incorporating information from both gene expression and cell clustering	Imputed expression matrix	NA	R	https://github.com/BMILAB/scHinter
MAGIC [81]	Expression matrix (e.g., CSV, TSV)	Uses Markov affinity-based graph imputation of cells, where data is imputed by DGE values across a graph representing cell similarities	Imputed expression matrix	Preserve local gene-gene interactions	Python	https://github.com/KrishnaswamyLab/MAGIC
**Batch-effect correction**						
Batchelor [82]	Expression matrix (e.g., CSV, TSV)	Uses methods like MNN to correct batch effects by aligning similar cells across batches	Batch-corrected expression matrix	Corrects batch effects while preserving biological variance	R	https://bioconductor.org/packages/release/bioc/html/batchelor.html
Beer [83]	Expression matrix (e.g., CSV, TSV)	Employs a two-stage model where data is modeled first and then batch effects are removed	Batch-corrected expression matrix	Integrates multiple datasets while reducing batch-related noise	R	https://github.com/jumphone/BEER/releases
Seurat [49]	Expression matrix (e.g., CSV, TSV)	Uses CCA and MNN to integrate datasets and correct for batch effects	Batch-corrected expression matrix, R objects	To integrate datasets from different conditions	R	https://satijalab.org/seurat/
scVI [84]	Expression matrix (e.g., CSV, TSV, HDF5)	Uses a VAE framework to model and correct batch effects by learning a latent space that separates biological signal from technical noise	Batch-corrected latent space, imputed matrix	Handling complex batch effects	Python	https://github.com/scverse/scvi-tools
MMD-ResNet [85]	Expression matrix (e.g., CSV, TSV)	Uses MMD combined with residual networks to align data across batches and remove batch effects	Batch-corrected expression matrix	NA	R	https://github.com/ushaham/BatchEffectRemoval
scScope [86]	Expression matrix (e.g., CSV, TSV)	Utilizes deep learning to learn a shared latent space that corrects batch effects while preserving biological variation	Batch-corrected expression matrix	NA	Python	https://github.com/AltschulerWu-Lab/scScope
Harmony [87]	Expression matrix (e.g., CSV, TSV)	Uses an iterative algorithm to align cell embeddings across batches while maintaining cell type structure	Batch-corrected embeddings	Preserve biological heterogeneity	R	https://github.com/immunogenomics/harmony
LIGER [73]	Expression matrix (e.g., CSV, TSV, HDF5)	Uses iNMF to jointly factorize data from multiple batches, effectively correcting for batch effects	Batch-corrected and integrated expression	Multi-omics data harmonization	R	https://github.com/welch-lab/liger
scBatch [88]	Expression matrix (e.g., CSV, TSV)	Uses a combination of clustering and empirical Bayesian methods to remove batch effects from single-cell RNA-seq data	Batch-corrected expression matrix	Correct batch effects while retaining meaningful biological signals	R	https://github.com/tengfei-emory/scBatch
**Clustering tools**						
SC3 [89]	Expression matrix (e.g., CSV, TSV)	Uses consensus clustering by combining results from multiple clustering methods to achieve robust cell groupings	Clustering labels, plots	NA	R	https://github.com/hemberg-lab/SC3
SCENIC [90]	Expression matrix, Gene Networks Regulatory (GRN)	Combines GRN inference with clustering, identifying co-expressed gene sets that drive cell type clustering	Cell clusters, regulatory network activity scores (e.g., CSV, RData)	Identifies regulatory networks and clusters cells based on gene regulatory interactions rather than direct gene expression	R	https://github.com/aertslab/SCENIC
BackSPIN [91]	Expression matrix (e.g., CSV, TSV)	Uses a biclustering approach that alternates between clustering genes and samples to achieve optimal partitions	Cell clusters, plots	Useful for complex cellular hierarchies	R	https://github.com/linnarsson-lab/BackSPIN
SIMLR [92]	Expression matrix (e.g., CSV, TSV)	Uses similarity learning to infer cell similarities and clusters by learning a low-dimensional space from the high-dimensional data	Clustering labels, latent embedding, plots	NA	R	https://github.com/BatzoglouLabSU/SIMLR
SAIC [93]	Expression matrix (e.g., CSV, TSV)	Iterative clustering method	Adjusted cell counts, cell clusters, plots	Ambient RNA correction	R	https://github.com/xiweiwu/SAIC.
GiniClust [94]	Expression matrix (e.g., CSV, TSV)	Gini index-based clustering	Cell clusters	Detects rare cell types	R and Python	https://github.com/lanjiangboston/GiniClust
RaceID [95]	Expression matrix (e.g., CSV, TSV)	k-medoids clustering, outlier detection	Cell clusters, CSV, RDS	Rare cell type identification	R	https://github.com/dgrun/RaceID
CIDR [96]	Expression matrix (e.g., CSV, TSV)	Clustering through imputation and dimensionality reduction	Clustering results, CSV, RDS	NA	R and C++	https://github.com/VCCRI/CIDR
GRACE [97]	Expression matrix (e.g., CSV, TSV)	Graph convolutional network (GCN) based clustering	Cell-type assignments CSV, HDF5	NA	R	https://github.com/th00516/GRACE
CosTaL [98]	Expression matrix (e.g., CSV, TSV)	KNN-graph based clustering	Corrected count matrix, CSV, HDF5	Removes technical variations	Python	https://github.com/li000678/CosTaL
DESC [99]	Expression matrix (e.g., CSV, TSV)	Deep learning based clustering	Cell clusters, CSV, RDS	NA	Python	https://eleozzr.github.io/desc/
scziDesk [100]	Expression matrix (e.g., CSV, TSV)	Deep learning (autoencoder-based)	Cluster assignments CSV, HDF5	NA	Python and R	https://github.com/xuebaliang/scziDesk
scVAE [101]	Count matrices	Variational autoencoder (VAE)	Latent representations CSV, HDF5	NA	Python	https://github.com/scvae/scvae
scDeep Cluster [102]	Count matrices	Deep learning-based clustering	Cluster assignments CSV, HDF5	NA	Python	https://github.com/keras-team/keras
SINCERA [103]	Expression matrix (e.g., CSV, TSV)	Utilizes hierarchical clustering and other standard clustering techniques to identify cell types and subtypes in single-cell RNA-seq data	Clustering labels, plots	Identify cell types and subtypes	R	https://research.cchmc.org/pbge/sincera.html
SEURAT [49]	Expression matrix (e.g., CSV, TSV)	Uses graph-based clustering, where cells are connected based on shared nearest neighbors, and clusters are identified as connected components in the graph	Clustering labels, UMAP/t-SNE embeddings, plots	NA	R	https://satijalab.org/seurat/
Monocle [104]	Expression matrix (e.g., CSV, TSV)	Uses a density peak clustering approach combined with pseudotime ordering to cluster cells and infer trajectories	Pseudotime trajectories, clusters, plots	NA	R	https://github.com/cole-trapnell-lab/monocle-release
SCRL [105]	Expression matrix (e.g., CSV, TSV)	Uses self-supervised learning to perform clustering in a reduced dimensional space, improving clustering accuracy	Clustering labels, plots	NA	C++	https://github.com/SuntreeLi/SCRL/
MultiK [106]	Expression matrix (e.g., CSV, TSV)	Uses k-means clustering on multiple data representations, combining results to identify robust clusters	Clustering labels, plots	Automates the selection of the optimal number of clusters for better accuracy	R	https://github.com/perou-lab/MultiK
Secuer [107]	Expression matrix (e.g., CSV, TSV)	Uses ensemble clustering methods to stabilize clustering results by combining multiple clustering algorithms	Clustering labels, plots	NA	Python	https://github.com/nanawei11/Secuer
Scanpy [50]	Expression matrix (e.g., CSV, TSV, H5 AD)	Uses Louvain or Leiden algorithms for clustering, which are graph-based methods that identify clusters based on cell-to-cell similarity networks	Clustering labels, UMAP/t-SNE embeddings, plots	NA	Python	https://scanpy.readthedocs.io/en/stable/
**Differential gene expression**						
Seurat [49]	Count matrix (e.g., CSV, TSV)	Uses a non-parametric Wilcoxon rank-sum test by default for DE analysis, with options for other statistical tests	Differential expression results (e.g., CSV)	NA	R	https://satijalab.org/seurat/
Monocle [104]	Count matrix, experimental design	Uses a GLM framework to identify genes that vary across cell states or pseudotime, often based on negative binomial distribution	Differential expression results (e.g., CSV), pseudotime analysis	Helps in studying dynamic gene expression changes during cell differentiation	R	http://cole-trapnell-lab.github.io/monocle-release/
MAST [108]	FASTQ, expression matrix (e.g., CSV, TSV)	Uses a hurdle model that combines a logistic regression model for zero inflation and a Gaussian linear model for positive counts	Differential expression results (e.g., CSV)	Handles dropout events and sparse data	R	https://github.com/RGLab/MAST
scDE [48]	Expression matrix (e.g., CSV, TSV)	Uses a Bayesian framework that models the dropout events and expression levels, distinguishing between technical noise and true differential expression	Differential expression results (e.g., CSV)	Reduce technical noise	R	https://www.bioconductor.org/packages/release/bioc/html/scde.html
EdgeR [109]	Expression matrix (e.g., CSV, TSV)	Statistical methods based on the negative binomial distribution, including empirical Bayes estimation, exact tests, generalized linear models, and quasi-likelihood tests	Differential expression results (e.g., CSV)	NA	R	https://bioconductor.org/packages/release/bioc/html/edgeR.html
DESeq2 [71]	Count matrix (e.g., CSV, TSV)	Uses a negative binomial GLM for count-based data, estimating dispersion and normalizing counts to perform DE analysis	Differential expression results (e.g., CSV)	NA	R	https://bioconductor.org/packages/release/bioc/html/DESeq2.html
Scotty [110]	Count matrix, experimental design	Allowing users to plan experiments by estimating the power to detect differential expression given certain parameters	Power analysis results, experimental design recommendations (e.g., CSV, TXT)	NA	R	https://github.com/mbusby/Scotty
Myrna [111]	FASTQ, expression matrix (e.g., CSV, TSV)	Uses a TMM normalization method and tests for differential expression using a modified version of the edgeR pipeline	Differential expression results (e.g., CSV)	Cloud-based	R	https://github.com/BenLangmead/myrna
GREIN [112]	Expression matrix (e.g., CSV, TSV)	Provides an online platform using a variety of methods (e.g., DESeq2, edgeR) for DE analysis	Differential expression results (e.g., CSV)	Web-based	R	http://ilincs.org/apps/grein/
D3E [113]	Expression matrix (e.g., CSV, TSV)	Statistical modeling of transcriptional dynamics to detect differential gene expression	Differential expression results (e.g., CSV)	NA	Python	https://hemberg-lab.github.io/D3E/

In droplet-based scRNA-seq protocols, another source of unwanted signals comes from ambient RNAs. These are RNA molecules that are freely floating in the cell lysate due to the breakdown of dead or dying cells before the droplets are separated. Since these ambient mRNAs are found everywhere, they add extra background noise and can greatly muddle the quality of the data and the true biological signals we are trying to capture [68]. Methods like SoupX, DecontX, and CellBender effectively remove ambient RNA influences in single-cell RNA-seq. SoupX uses known negative markers (genes not expressed in specific cell types), while DecontX employs Bayesian inference, and CellBender uses deep generative models [63, 68].

Low-abundance genes should be excluded as they do not provide enough information for reliable analysis. Additionally thresholds should be set based on the number of cells expressing a gene or the genes average expression level [18, 45, 65]. Depending on the analysis, non-coding genes may be excluded to simplify the data. In scRNA-seq data, mitochondrial genes are discarded after quality control to avoid biases, as mitochondrial transcripts are not usually expressed in the nucleus [114, 115].

Among quality-checking and preprocessing tools, Solo was reported to have an accuracy of 83.59%, with a runtime of 13–17 min and memory usage of 7–12 GB across different standard datasets [61]. Scrublet demonstrated an accuracy of 99% and supported scalability but lacked data on runtime and memory consumption [52]. DropletQC can process 100 million reads in under 133 s on 8 CPUs with 16 GB RAM, highlighting its efficiency for nuclear fraction analysis [60]. ScPipe required 10 h to process a dataset with 112 million reads while consuming 540 GB of RAM, making computation highly resource-intensive [66]. CellBender exhibited adaptability, with runtime varying from 20 min to 1 h depending on dataset size and GPU availability, though precise memory usage data was unavailable [64]. DoubletFinder was notable for its limitation in detecting homotypic doublets and the need for parameter optimization, but it performed better when integrated with sample multiplexing [67].

### Normalization

Normalization is an important process in scRNA-seq data analysis, as it helps focus on meaningful information by fixing issues like differences in how well genes are captured, the depth of sequencing, and other technical variations that can affect the data. There are two main categories of normalization: within sample normalization and between sample normalization [12, 116] (Table 4). Within-cell normalization methods like calculating TPM (Transcripts Per Kilobase Million) or RPKM (Reads Per Kilobase of transcript per Million mapped reads)/FPKM (Fragments Per Kilobase of transcript per Million mapped reads) are commonly used to address sequencing depth within individual cells. But, these methods may not be suitable for certain downstream analyses as they do not account for changes in RNA content and can be misleading when analyzing differentially expressed genes. However, studies in bulk RNA-seq have emphasized the vital role of between-sample normalization. The non-linear normalization method, without UMIs, effectively explores cellular heterogeneity and accurately analyzes scRNA-seq data with high library sizes. This method computes individual normalization factors for each cell and gene by using information from multiple genes and cells, reducing technical biases in scRNA-seq. It is more flexible than traditional size factor normalization, as it estimates using many genes with minimal constraints [117]. For example, DINO works on the principle of non-linear method [69].

In scRNA-seq normalization, two main methods are employed: cell-based and gene-based. The cell-based approach calculates a specific size factor for each cell, used to normalize its gene expression. “Scran” uses this by pooling cells for robust size factor estimation and reducing the impact of excessive zeros. On the other hand, gene-based methods like SCnorm and SCTransform in Seurat adjust genes based on their sequencing depths or abundance levels. This distinction enables precise normalization in scRNA-seq analysis [45, 72, 118, 119].

Among normalization tools for scRNA-seq, Scanpy and Seurat normalization stands in terms of scalability [49, 50]. DESeq2, which is a differential expression tool, has an inbuilt method for normalization and presents concern with false positive rates, which can affect downstream analysis [120]. LIGER is a very useful tool but has limitations in integrating diverse features like gene expression and intergenic methylation, potentially reducing its effectiveness for multiomics studies [73].

### Imputation method

ScRNA-seq data often exhibit missing values, notably zero expression counts for numerous genes. Some of these zeros have biological significance, indicating either gene inactivity or mRNA degradation post-expression. Additionally, technical and sampling factors in scRNA-seq contribute to non-biological zeros, arising from issues like reverse transcription failures, low mRNA quantities, inefficient amplification, or restricted sequencing depth. These non-biological zeros introduce intercellular variability, impede the detection of gene relationships, and exert a notable influence on downstream analyses. In contrast, employing imputation, i.e., replacing missing values with estimated alternatives, proves an effective strategy for addressing these missing data.

Effectively classifying and comparing popular imputation methods is paramount in providing users with informed guidance for various datasets and unique needs (Table 4). These methods can be broadly categorized into four distinct groups:

- Model-based methods rely on statistical models encompassing technical and biological variability, estimating parameters to perform imputation.
- Low-rank matrix-based approaches utilize a low-rank matrix to uncover spatial representations of cells, capturing linear relationships and reconstructing a less sparse expression matrix.
- Data smoothing methods, on the other hand, leverage gene expression values from similar cells to adjust all values, including zeros and non-zeros, employing a smoothing technique.
- Deep learning methods use advanced techniques to identify potential spatial representations of cells and reconstruct the observed expression matrix based on these estimated representations. This classification provides users with a structured framework for selecting the most suitable imputation method tailored to their specific dataset and analytical requirements [121].

Ruochen Jiang stresses the importance of tailoring imputation methods to the specific attributes of single-cell data [122]. For UMIs-based sequencing, which lacks zero-inflation, using imputation methods designed for zero-inflated models is inappropriate. While tasks like cell dimensionality reduction or clustering can often be performed without imputation at the cell level, selecting the correct imputation method is critical for optimal performance in differential expression (DE) analysis. Conversely, non-UMI data benefits from imputation utilizing a non-zero inflation model, particularly for tasks like cell down scaling or clustering. In DE analysis, any imputation method outperforms no imputation or binarization. Ultimately, in cases where the cell library is sufficiently extensive, indicating ample sequencing depth, imputation may be unnecessary. This underscores the importance of aligning imputation strategies precisely with the distinctive characteristics and analytical objectives of single-cell datasets [12].

IGSimpute offers GPU-accelerated imputation but is unsuitable for rare cell types, with training times ranging from 4 min (100,000 cells) to 64 min (1,000,000 cells) using a batch size of 1000 [74]. SciGAN requires large datasets with at least a few thousand training samples but lacks detailed runtime or scalability data [75]. CMF-Impute may introduce bias when dropout events are abundant, with runtimes ranging from 6 to 12.6 min (0.21 hours), depending on dataset size [123]. DrImpute, achieving an accuracy of 96%, focuses on cell-level correlations but ignores gene-level correlations, limiting its precision. It requires approximately 750 s for 10,000 cells [78]. DeepImpute shows a low mean squared error (MSE = 0.0259) and high correlation (0.984) with ground truth, making it a robust option, running in about 12 min on a dataset with 50,000 cells using 10 GB of RAM on an 8-core machine [76]. ScLRTC provides clustering performance indicators (ARI = 0.7, NMI = 0.8) and takes approximately 8000 s to process 12,500 cells [79]. ScHinter offers a high ARI of 0.9 and a highly variable runtime, from 0.3 to 56.22 s, depending on dataset complexity [80].

The choice of an imputation tool depends on dataset size, computational constraints, and the level of accuracy needed, with DeepImpute, DrImpute, and ScHinter appearing as strong contenders for balancing accuracy and efficiency.

### Batch effect

Variations in single-cell RNA-sequencing data are known to be influenced by technical factors. In some cases, the measurement of biological variations among the samples is affected by these technical factors, making it difficult to address the research problems. Confounding factors in single-cell RNA-sequencing data encompass experimental biases and batch effects. Systematic technical biases, like unequal PCR amplification, cell lysis discrepancies, variable reverse transcriptase enzyme efficiency, and stochastic molecular sampling during sequencing, are unavoidable sources of variation [124] (Table 4). Therefore, batch effect is the major challenge that needs to be resolved before downstream analysis.

Batchelor demonstrates a linear increase in CPU time with dataset size, requiring 2 min for 7000 cells and up to 20 min for 70,000 cells, indicating scalability but potential inefficiencies for extremely large datasets [82]. Beer is a lightweight tool with a runtime of only 1–5 min but lacks further performance details [83]. scVI completes batch correction on 100,000 cells in 25 min, leveraging an NVIDIA Tesla K80 GPU with 24 GB RAM, making it suitable for large-scale analyses [84]. scScope takes under 100 min for 8000 cells across five iterations on a high-performance Xeon E5 CPU with 64 GB RAM and an Nvidia Titan X GPU, suggesting it is computationally demanding [86]. Harmony processes 500,000 cells in 68 min while consuming 7.2GB RAM, offering a balance between efficiency and resource usage [87]. LIGER provides batch correction but has a limitation in multiomics integration, which may affect studies requiring diverse data integration [73]. scBatch is not recommended for highly imbalanced study designs and exhibits scalability issues, handling a few hundred cells in minutes but taking hours for datasets exceeding 1000 cells [88].

The choice of a batch correction tool depends on dataset size, computational resources, and integration requirements, with Harmony and scVI standing out for large-scale studies, while Beer offers a quick but less detailed solution.

### Feature selection and dimensionality reduction

In managing high-dimensional data, dimensionality reduction stands out as a crucial strategy, alongside feature selection (Table 4). When dealing with single-cell RNA-sequencing data, a dual-step approach is often required. Initially, principal component analysis (PCA) is employed to simplify the data. Subsequently, techniques like t-distributed stochastic neighbor embedding (t-SNE) or Uniform Manifold Approximation and Projection (UMAP) are utilized to create visual representations for enhanced comprehension [3]. PCA, a potent mathematical tool, adeptly handles large datasets, preserving both local and long-range patterns. Each principal component acts as a unique axis, orthogonal to the others, enabling the reconstruction of the overall genetic makeup. Determining the number of principal components involves identifying the top ones explaining 80 to 90% of the total variances, or discerning an “elbow point” in the analysis [125].

To address missing or dropout data readings, adaptations of PCA have emerged, incorporating the zero-inflated negative binomial distribution (ZINB) [126]. t-SNE, a non-linear technique, excels in preserving local relationships among data points, effectively segregating clusters. Nevertheless, it may not accurately represent long-range relationships or structures in the data [125, 127]. Diffusion map (DM), another widely used non-linear technique, condenses both nearby and far-reaching patterns into a lower dimension, specially designed to track subtle shifts and transformations in a transcriptome [128]. UMAP, a computationally efficient method, surpasses DM and t-SNE. It captures both local and long-range patterns, recovering global structures in single-cell RNA-sequencing data [129]. Non-linear projection techniques like DM, t-SNE, and UMAP can compress data into 2 or 3 dimensions, but they may introduce distortions and non-biological artifacts, making them primarily recommended for visualization [11].

### Cell clustering

ScRNA-seq clustering helps elucidate cell-to-cell heterogeneity and uncover cell sub-groups and cell dynamics at the group level. Different methods have been created to find different types of cells in single-cell RNA-seq data (Table 4). There are five methods of clustering: K-means clustering, hierarchical clustering, graph based clustering, density-based clustering, and deep learning-based clustering [130]. K-means clustering is a widely used method for grouping data. It works by repeatedly finding a set number of cluster centers (called centroids) in a way that minimizes the total squared distance between each data point and its closest centroid. This method is efficient even with large datasets, as it scales well with the number of data points [131]. SAIC and RaceID both the clustering tools are based on k-means clustering [93, 95].

Hierarchical clustering is widely used in single-cell RNA-seq analysis. It comes in two types: agglomerative, where cells merge based on similarity, and divisive, where clusters are recursively split. These strategies form a hierarchical structure, aiding in identifying rare cell types. Unlike some methods, hierarchical clustering does not require any pre-determined number of clusters, or make assumptions about data distributions. Thus, many single-cell RNA-seq clustering methods include hierarchical clustering [4]. CIDR, BackSPIN, and SINCERA are the clustering tools which are based on hierarchical clustering [91, 96, 103].

DBSCAN is a popular density-based clustering algorithm capable of identifying clusters with arbitrary shapes and outliers. Unlike many clustering methods, DBSCAN does not require the pre-specification of the number of clusters. However, it demands users to set two parameters: $\epsilon$ (eps) and the minimum number of points (minPts) to define dense regions that influence DBSCAN clustering [132]. GiniClust and Monocle2 are two tools which are based on DBSCAN clustering [130].

Graph-based clustering, also known as community-based clustering, plays a crucial role in disciplines like sociology, biology, and systems analysis. It is particularly applicable to scenarios represented as interconnected nodes and edges. In single-cell RNA-seq data, nodes represent cells, and connections are determined by pairwise cell-cell distances. The approach involves isolating the branch with the highest weights (cell-to-cell distances) in a dense graph, reflecting cellular relationships. The three primary methods for community detection-based clustering are the clique algorithm, spectral clustering, and the Louvain algorithm [133]. GRACE and CosTaL are well known graph based clustering tools [98, 134].

This deep learning model utilizes a denoising autoencoder to reconstruct uncorrupted data from intentionally corrupted inputs, enabling robust handling of noisy observations. By introducing random Gaussian noise, it simulates minor data variations. The encoder and decoder functions, implemented with rectifier-activated neural networks, process the corrupted input [102]. DESC, scziDesk, scVAE, and scDeepCluster are well known methods that come under deep learning clustering [102, 130]. The evaluation of clustering performance commonly relies on metrics like adjusted R and index for correctness, normalized mutual information (NMI) and Jaccard index for similarity, and Silhouette coefficient and Dunn index for compactness and separateness of clusters [12].

scRNA-seq analysis tools offer diverse capabilities, each with strengths and limitations in terms of computational efficiency, scalability, and sensitivity. SCENIC demonstrates high specificity (0.99) and sensitivity (0.88) for cell-type identification but demands significant memory (128 GB) [90]. SIMLR and Monocle provide efficient analysis times, with Monocle processing 8365 cells in 9 min, making them suitable for rapid computations [92]. DESC achieves high clustering accuracy (adjusted Rand index of 0.919–0.970) and efficiently processes large datasets (30,000 cells) using a NVIDIA TITAN Xp GPU [100]. However, RaceID and CIDR have limitations, with RaceID showing reduced sensitivity to low-expressed genes and CIDR’s accuracy decreasing with higher dropout rates [96, 97]. MultiK excels in identifying rare cell populations, even as small as 0.5%, but is computationally expensive [106]. Secuer outperforms traditional clustering methods, being five to twelve times faster than k-means and Louvain/Leiden, making it highly suitable for ultra-large datasets [107]. scDeepCluster scales well for up to 100,000 cells but requires substantial computational resources [102].

Ultimately, tool selection depends on dataset size, computational constraints, and the need for rare cell-type identification, making comparative evaluation crucial for optimizing scRNA-seq analysis.

### Differential expression (DE) analysis

DE analysis is crucial for identifying genes that have significant differences in expression levels between distinct subpopulations, groups of cells, or under specific disease conditions in single-cell RNA-sequencing experiments [135, 136]. Differentially expressed genes (DEGs) play a vital role in understanding the biological differences between compared conditions [120]. Cell states within a population lead to unique gene expression patterns, and data processing methods have considerable impact on the analysis of differential expression, enabling the evaluation of their performance using the results of the analysis [12].

DESeq2 and EdgeR, initially developed for bulk RNA-seq experiments, are also widely utilized in single-cell RNA-seq studies [71, 109]. DESeq2 employs a generalized linear model (GLM) for each gene, incorporating shrinkage estimation for stabilizing variances and fold changes. It applies statistical tests like the Wald test or likelihood ratio (LR) test to assess significance [71, 137]. In contrast, EdgeR fits a GLM with negative binomial (NB) noise for each gene, estimates dispersions using conditional maximum likelihood, and employs a tailored exact test suitable for over dispersed data to identify DEGs [109, 137].

Various methods have emerged to address challenges posed by dropouts and the presence of multiple expression modes in single-cell RNA-sequencing data analysis (Table 4). For example, MAST utilizes a GLM and accounts for dropouts by fitting them to a bimodal distribution, while Monocle incorporates a Tobit model to address dropout events and employs a generalized additive model (GAM) for effective data fitting [108]. SCDE models gene expression as a combination of Zero-Inflated Negative Binomial (ZINB) distributions and uses Bayesian techniques to estimate posterior probabilities for differentially expressed (DE) genes [48]. D3E introduces a novel perspective by modeling the distribution of gene expression through the bursting model of transcriptional regulation. scDD employs a multi-modal Bayesian modeling framework to capture the diverse distributions found in single cells, providing a versatile solution in this complex field of study [45]. Recently, Soneson and Robinson conducted a comprehensive assessment of [36] DE methods, including those designed for both single-cell RNA-seq and bulk RNA-seq data. Their evaluation highlighted substantial differences among these approaches, particularly in terms of the characteristics and quantity of identified DEGs [138]. As the field continues to advance, an increasing number of tools dedicated to the analysis of differential expression in single-cell RNA-sequencing data will be developed. In order to accurately identify DEGs, it is important to select tools specifically designed for scRNA-seq.

Monocle demonstrates the greatest sensitivity (0.765) but also generates a high number of false positives, making it less reliable for certain datasets [104]. MAST, on the other hand, offers high precision but lower sensitivity (0.198) and struggles with highly multi-modal data [108]. EdgeR and DESeq2, originally designed for bulk RNA-seq, achieve intermediate sensitivity (0.58 and 0.695, respectively), but they may not optimally handle zero counts or multi-modality in scRNA-seq [71, 109]. However, DESeq2 performs better than EdgeR, with a higher true positive rate (TPR). D3E shows high sensitivity (0.722) but also introduces false positives [113]. Scotty focuses on optimizing experimental design but may introduce bias against genes with low read counts [110]. GREIN lacks functionalities for downstream analyses, while Myrna lacks available data [111, 112]. Overall, the choice of tool depends on the balance between precision, sensitivity, and computational trade-offs in the analysis of scRNA-seq data.

### Further analysis step

Following differential expression analysis and clustering, several downstream analyses can provide deeper insights into cellular mechanisms. Trajectory inference is employed to map cell differentiation processes, elucidating the progression of cellular states. Cell-cell communication analysis, based on ligand-receptor interactions, helps in understanding intercellular signaling networks. Gene regulatory network construction enables the identification of key transcriptional regulators governing gene expression. Additionally, pathway and functional enrichment analysis facilitates the identification of critical biological pathways associated with cellular functions and disease mechanisms. Furthermore, metabolic and functional state analysis provides a comprehensive view of cellular metabolism and functional alterations, offering insights into disease progression and potential therapeutic targets.

## Single cell databases

There are multiple databases which offer invaluable resources for researchers delving into various facets of single-cell transcriptomics (Table 5). scRNASeqDB is a repository housing 38 human single-cell transcriptome datasets, which provides researchers access to gene expression profiles across 200 distinct cell types, totaling 13,440 samples [6]. TMExplorer, on the other hand, specializes in TME scRNA-seq datasets, providing access to 48 datasets representing 28 different cancer forms [139]. scREAD is a pivotal resource for Alzheimer’s disease research, offering access to 73 datasets across 10 brain regions, providing researchers with information on cell-type predictions and DGEs analyses [140]. SC2 disease is a curated database for exploring cellular heterogeneity across diverse cell types in various diseases, containing 9,46,481 entries categorized into 341 specific cell types, 29 distinct tissues, and 25 different diseases [141]. PlantscRNAdb uniquely focuses on plant species, featuring 26,326 marker genes spanning 128 distinct cell types [142]. EndoDB specializes in endothelial cells, providing curated data from 360 datasets, comprising 4741 bulk and 5847 single-cell endothelial transcriptome [143]. Lastly, SCAD-Brain integrates data from 17 projects related to Alzheimer’s disease, encompassing 21 datasets with 359 samples, enabling analyses such as cell marker analysis, gene expression analysis, and pathway enrichment [143]. These databases collectively provide crucial information on cellular heterogeneity, gene expression profiles, and disease-specific transcriptomic patterns.

**Table 5:** Databases for scRNA-seq studies and their summary

Database name	Host institute	Nature of database	Link
scRNAseqDB [6]	School of Biomedical Informatics and School of Public Health, University of Texas Health Science Center, USA	Human single cell gene expression datasets	https://bioinfo.uth.edu/scrnaseqdb/
scREAD [140]	Bioinformatics and Mathematical Biosciences Lab, The Ohio University, Ohio	Alzheimer’s disease dataset	https://bmbls.bmi.osumc.edu/scread/
SC2 disease [141]	School of Computer Science, Northwestern Polytechnical University, China	Comprehensive datasets	http://easybioai.com/sc2disease/
DRscDB [144]	DRSC-Harvard Medical School, USA	Comprehensive datasets	https://www.flyrnai.org/tools/single_cell/
PlantscRNAdb [142]	Institute of Crop Sciences/Institute of Bioinformatics, Zhejiang University, China	Plant dataset	http://ibi.zju.edu.cn/plantscrnadb/
EndoDB [143]	Carmeliet Lab, VIB - KU Leuven Center for Cancer Biology, Belgium	Endothelial cell transcriptomics data	https://vibcancer.be/software-tools/endodb
SCAD-Brain [145]	Hu Lab, School of Medicine, WUST, China	Datasets of human and mouse brains with Alzheimer’s disease	https://www.bioinform.cn/SCAD/
PanglaoDB [146]	Integrated Cardio Metabolic Centre Karolinska Institutet, Blickagången 6, 141 57 Huddinge, Sweden	Comprehensive datasets	https://panglaodb.se/index.html
Single Cell Expression Atlas	EMBL-EBI	Comprehensive datasets	https://www.ebi.ac.uk/gxa/sc/home
Single Cell Portal [147]	Broad Institute of MIT and Harvard	Comprehensive datasets	https://singlecell.broadinstitute.org/single_cell
CELLxGENE [148]	Chan Zuckerberg Initiative, 1180 Main Street, Redwood City, CA 94063, USA	Comprehensive datasets	https://cellxgene.cziscience.com/
Allen Brain Cell Atlas	Allen Institute for Brain Science	Mammalian brain	https://portal.brain-map.org/atlases-and-data/bkp/abc-atlas
CellMarker 2.0 [149]	College of Bioinformatics Science and Technology, Harbin Medical University	Comprehensive datasets	http://bio-bigdata.hrbmu.edu.cn/CellMarker/

## Current research and gaps

With the help of scRNA-seq, data analysis at single-cell resolution is possible to some extent and is expected to advance in the future, proving to be a vital technique for data analysis. These techniques rely on identifying cellular differences, understanding their communication, and recognizing unique or rare cellular states. We will discuss these approaches to scRNA-seq, how they can be implemented in various research domains, and provide some examples of their applications.

### Cellular heterogeneity

ScRNA-seq is a technique used to explore single-cell expression among a population of cells, characterizing cellular heterogeneity. It identifies unique gene expression profiles, highlighting specific cells that can serve as biomarker for disease diagnosis [150]. Additionally, it can reveal significant transcriptomic changes in disease individuals compared to a healthy ones, suggesting their role in disease progression [151]. For example, different transcriptional profiles in tumors identify immune-evading clones, drug-resistant subpopulations, and cancer stem-like cells, all of which aid in the advancement of the disease and resistance to treatment. Functional heterogeneity among T cells, B cells, and myeloid cells has been revealed by scRNA-seq in the immune system, outlining how distinct immune cells react to infections, inflammatory cues, and antigenic challenges. Therefore, scRNA-seq thoroughly examines important gene expression patterns and uncovers significant biomarkers, receptors, ligands, and transcription factors, which lay the foundation for the functional analysis of cells [75].

### Cell-cell communication

The utilization of single-cell RNA-sequencing data for the purpose of examining cell-to-cell communication is a powerful methodology for elucidating inter-cellular communication pathways. Nevertheless, prevailing approaches commonly do this analysis by focusing on cell categories or clusters, disregarding the intricate details at the level of individual single cells. A novel approach introduced for analyzing interactions at the single-cell level was Scriabin [152].

Using this method, scientists can map cellular interactions in real time and pinpoint biologically important pathways across a range of tissues. Scriabin has been utilized in immuno-oncology to analyze immune-tumor crosstalk and identify ligand-receptor interactions that promote immune evasion. Precision immunotherapies have been made possible by the discovery of novel checkpoint inhibitors and tumor-supporting stromal signals by researchers using scRNA-seq data analysis. It employs a combination of curated ligand-receptor interaction databases [153, 154], models of downstream intracellular signaling [155], anchor-based dataset integration [49], and gene network analysis [156] to examine intricate communication pathways at the resolution of individual cells. This approach allows for the identification of biologically significant connections between cells at a single-cell level.

### Cell type identification

ScRNA-seq provides a valuable avenue for the complete sequencing and annotation of cell types within various tissues of a given species [156–158]. This technique facilitates the identification of both known and novel cell types, hence enabling an understanding of their associated biological processes and molecular activities. For example, in a sample size of around 25,000 bipolar cells in mice, researchers found two distinct types of unique mouse retinal bipolar cells. Notably, one of these cell types had a shape that deviated from the conventional structure often observed in bipolar cells [159]. Furthermore, significant cellular heterogeneity within the retinal bipolar cell population was revealed by scRNA-seq. As a result, using computational clustering and differential gene expression analyses, researchers were able to identify hidden molecular signatures associated with these morphologically distinct bipolar cells. This method identified a type of atypical bipolar cells that might have important effects on visual processing by playing particular roles in retinal signaling pathways. One excellent example of how scRNA-seq can be used to enhance cellular classifications and find new targets for additional research into retinal development and related visual disorders is this kind of characterization. Moreover, computational approaches for cell type detection do not need manual annotation. Alternatively, these tools may be utilized to make direct predictions of cell types based on publicly available resources of scRNA-seq data [160].

## Applications

As we discussed about current research in scRNA-seq, it offers plenty of implementation in various domains. It includes drug discovery, microbial profiling, tumor study, stem cell research, and so on. Here, we will describe the importance and how it can be implemented. The following are the applications.

### Drug discovery and development

Since the introduction of whole-transcriptome profiling of a single cell in 2009 [4], this technology has evolved enormously by giving results at the single-cell level. One such example is drug discovery and development, in which scRNA-seq investigations were carried out by Van de Sande et al. [161] on brain tissues obtained from both healthy mice and mouse models of Alzheimer’s disease, which revealed the presence of disease-associated microglia. There are differential expression patterns seen in such microglia clusters that point to novel molecular targets that might be taken advantage of to control negative neuroinflammation reactions. The study revealed evidence that particular microglial subsets are preferentially activated under disease conditions, implying that focused treatments could specifically target these pathogenic populations. This high resolution data made possible by scRNA-seq which highlights the need of spotting rare cell types possibly crucial for the course of neuro-degeneration. These findings indicate that a therapeutic approach targeting specific cell states may hold potential benefits for those afflicted with Alzheimer’s disease. In the end, scRNA-seq enables the early identification and characterization of therapeutic targets linked to diseases. Early detection of potential issues can ultimately decrease the occurrence of clinical failures, hence, enhancing the efficiency of the drug development process.

### Tumor microenvironment (TME)

ScRNA-seq is a very powerful technology that enables the study of heterogeneous single-cell populations in a TME [162, 163]. Moreover, it gives clarity to marked differences among cells in a cell population. It allows for the comprehensive examination of gene expression patterns in individual cells, which may not be readily apparent in bulk analysis. Furthermore, throughout a study, it enables researchers to identify and analyze the diverse cellular composition within a TME. In the study of glioma, Li et al. identified 14 glioma cellular sub-populations and 7 primary cell types [164], demonstrating the intricate and varied nature of the TME. Since the different cell types exhibit different gene expression patterns, metabolic adaptations, and immune evasion strategies, this heterogeneity is absolutely important in determining tumor evolution and response to treatment. The study also highlighted how interactions between glioma cells and immune components such as regulatory T cells and tumor-associated macrophages (TAMs) create an immunosuppressive environment that promotes tumor growth. Glioma cells also show metabolic plasticity, meaning they can change between glycolysis and oxidative phosphorylation depending on microenvironmental signals, enabling their adaptation to changes in therapeutic pressure and nutrient availability. Another important discovery was the identification of specific molecular markers and signaling pathways linked to several glioma subtypes, thus providing possible targets for precision treatments. Another study conducted by Ding et al. from Harvard Medical School utilized single-cell profiling techniques to investigate the presence of sub-clonal heterogeneity and identify aggressive disease states in triple-negative breast cancer (TNBC) [165]. Initially, on untreated TNBC tumors, the investigators confirmed the presence of cellular heterogeneity within primary TNBCs with the help of scRNA-seq. Furthermore, employing clustering methods, they have successfully identified five discrete clusters of cells. As this method provides more diverse cells, it is suitable for evaluating cellular heterogeneity in TME. Thus, scRNA-seq could be an appropriate platform for research on TME [166].

### Biomarker discovery

Biomarker discovery is a crucial step for disease diagnosis, and it can be effectively carried out using scRNA-seq analysis. This technology allows for the identification gene biomarkers, their regulatory factors, and their signaling pathways involved in disease mechanisms [167]. In a study of clear cell renal cell carcinoma (ccRCC) [168], Narayanan et al. identified SLC6 A3 as a potent diagnostic and prognostic biomarker using scRNA-seq, paving a way for improved diagnosis and treatment strategies. Its specificity for malignant tissues is demonstrated by the fact that SLC6 A3 expression is markedly increased in ccRCC tumor cells but absent in immune cells and benign kidney tubules. This finding was confirmed by a number of datasets, such as scRNA-seq profiles, microarray datasets (GSE40435, GSE53757), and TCGA pan-cancer expression data, thereby confirming its potential as a diagnostic marker. SLC6 A3 is a promising candidate for early disease detection because of its high sensitivity and specificity in differentiating ccRCC from normal kidney tissue, as further demonstrated by receiver operating characteristics (ROC) analysis. Therefore, it is important to utilize this technique in biomarker discovery, as it plays a vital role in uncovering the molecular mechanism of diseases and determines strategies for tackling them [169].

### Microbes profiling

Different subpopulations of bacteria within a community can exhibit diverse gene expression patterns and dynamically adjust to challenging conditions. This expression heterogeneity, which is prevalent in natural micro-biota, is difficult to capture using bulk sequencing approaches. However, microbes present challenges, such as cell size and cell wall variation, as well as low of mRNA content per cell, and the absence of poly(A) tails in mRNA. To address these issues, Pu et al. developed a novel method called Ribosomal RNA-derived cDNA Depletion (RiboD), integrated into the PETRI-Seq technique, to capture single-cell transcriptomes of Gram-positive and Gram-negative bacteria with high purity and low bias [170, 171]. This method, known as RiboD-PETRI, offers a high-throughput, cost-effective solution for bacterial scRNA-seq. It allows precise exploration of bacterial population heterogeneity in biofilms and microbiomes revealing subpopulations with distinct gene expression profiles that influence the dynamics and behavior of biofilms communities. Using RiboD-PETRI, scientists can now see microbial communities more clearly and thoroughly, especially in settings like microbiomes and biofilms where cellular heterogeneity is essential for adaptation and survival. By identifying transcriptional alterations that reflect metabolic changes, the emergence of antibiotic resistance, and the regulation of quorum sensing, the method makes it easier to identify functionally distinct bacterial subpopulations within biofilms. A deeper understanding of how subpopulations contribute to community structure and function is made possible by scRNA-seq with RiboD-PETRI, which reveals rare phenotypic variants in contrast to bulk sequencing, which averages gene expression across a population. These findings highlight the importance of understanding microbial heterogeneity in developing therapeutics [170].

### Stem cell research

The use of single-cell sequencing technology offers distinct advantages in comprehending the occurrence and progression of stem cells. In the course of stem cell growth, there exists temporal variation in gene expression, posing challenges that have been arduous to address using conventional methodologies. The utilization of single-cell sequencing enables researchers to direct their attention toward a solitary cell, whether it is seen as an independent entity within a larger cell population or as a representative of a certain subpopulation across several developmental phases. The integration of single-cell sequencing with other sophisticated methodologies holds great potential for further enhancing scientific inquiry. For instance, the combination of scRNA-seq with patch-clamp technology presents an intriguing avenue for investigating the underlying mechanisms of neuropsychiatric disorders, therefore unraveling its fundamental essence [172].

Human primordial germ cells (hPGCs) serve as the progenitors for fully developed germ cells. The transcriptomes of hPGCs at the single-cell level exhibit a notable degree of homogeneity during both the migration and gonad phases [173]. Li et al. endeavored to elucidate the trajectory of development and variability of fetal female germ cells [174]. A comprehensive analysis was conducted on over 2000 germ cells and their corresponding gonadal niche cells using scRNA-seq across many developmental stages. The primary findings of this investigation encompass the identification of distinct transcriptome attributes shown by transcription factor networks across several stages of development. It allows researchers to identify gene regulatory networks and stage specific transcription factors that govern the transition from pleuripotency to lineage commitment. The study shed light on the sequential activation of important pathways involved in germ cell maturation by identifying different transcriptomic signatures in early migrating hPGCs compared to those that had reached the gonadal ridge. Additionally, the use of scRNA-seq has revealed previously unexplored subpopulations in the germline during development, emphasizing the existence of transcriptionally unique cells that might have specialized functions in gametogenesis.

## Conclusion

In conclusion, the evolution of scRNA-seq has revolutionized our understanding of cellular complexities and heterogeneity, paving the way for advanced research across various biological landscapes. With the establishment of novel methodologies, tools, and databases, researchers can now delve deeper into the mechanisms governing gene expression at an individual cell level, addressing pivotal challenges in drug discovery, TMEs, and cellular communication. As we continue to explore the vast potential of scRNA-seq, it becomes increasingly essential to adopt tailored computational tools that enhance data accuracy, mitigate biases, and refine analysis techniques. By harnessing the latest advancements and remaining cognizant of existing gaps, the scientific community can leverage scRNA-seq to uncover critical biological insights, ultimately driving forward our understanding of health and disease in unprecedented ways.

## Data Availability

No datasets were generated or analyzed during the current study.
